# Supplementary material for: Estimands and Cumulative Incidence Function Regression in Clinical Trials: Some New Results on Interpretability and Robustness
Source: Stat Med. 2024 Oct 29;43(29):5513–33. doi: 10.1002/sim.10236 (PMC11589047; doi:10.1002/sim.10236)
Supplement: Supplementary file 1 — Data S1. Supporting Information. [file SIM-43-5513-s001.pdf]

Received: Added at production | Revised: Added at production | Accepted: Added at production

DOI: xxx/xxxx

SUPPLEMENTARY MATERIAL

Estimands and cumulative incidence function regression in clinical trials: some new results on interpretability and robustness

Alexandra Bühler | Richard J. Cook | Jerald F. Lawless

This document provides additional results for the numerical investigation in Section 3.2 and the simulation study in Section 4.2.

S1 | LIMITING VALUES OF FG AND DB TREATMENT EFFECT ESTIMATORS UNDER INTENSITY-BASED PROCESSES

S1.1 | Supplementary results for the numerical investigation in Section 3.2

In this section we report on additional results about the estimands arising from Fine-Gray (FG) and direct binomial (DB) regression when the true process is governed by time-homogeneous intensities of proportional form as in Section 3.2. Figure S1 illustrates the dependency of $\exp(\beta^{\star}_{FG})$ and $\exp(\beta^{\star}_{DB})$ on $\exp(\gamma_2)$ for different values of $\exp(\gamma_1)$ and $P(T_1 < T_2 | T \leq 1, X = 0)$. Figure S2 depicts the limiting values $\exp(\beta^{\star}_{FG})$ and $\exp(\beta^{\star}_{DB})$ as a function of $P(T_1 < T_2 | T \leq 1, X = 0)$ for different values of $\exp(\gamma_1)$ and $\exp(\gamma_2)$; a discussion of results can be found in Section 3.2.
The plots in Section 3.2 and Figures S1-S2 give insights into how the limiting values $\beta^{\star}_{FG}$ and $\beta^{\star}_{DB}$ of the FG and DB estimators vary with the parameters of the intensity-based process, and in particular how they compare with $\exp(\gamma_1)$, the treatment effect on the main $0-1$ intensity. Another question of interest concerns the effect of model misspecification on the estimation of $F_1(t|X)$. Specifically we are interested in how $F_1(t|X)$ (c.f. (2.4)) compares with $F^{\star}_{1,FG}(t|X)$ and $F^{\star}_{1,DB}(s_r|X)$ as given by (3.10) and (3.17), respectively. Plots of $F_1(t|X)$ versus $F^{\star}_{1,\cdot}(t|X)$ are depicted in Figure S3 for $\exp(\gamma_1) = 1$ and in Figure S4 for $\exp(\gamma_1) = 0.75$ when $P(T_1 < T_2 | T \leq 1, X = 0) = 0.6$ (upper panels) and 0.2 (lower panels). We find that $F^{\star}_{1,\cdot}(t|X)$ under either estimation procedure is closer to the truth $F_1(t|X)$ for values of $\exp(\gamma_2)$ close to 1 and as $P(T_1 < T_2 | T \leq 1, X = 0)$ increases.

S1.2 | Limiting values under time-inhomogeneous intensities

Here we assume a competing risks process with Weibull intensities $\lambda_{0k}(t|X) = \lambda_k(t)\exp(\gamma_k X) = \kappa_k \lambda_k (\lambda_k t)^{\kappa_k - 1}\exp(\gamma_k X)$, $k = 1, 2$, and investigate how time-inhomogeneous intensities may influence the limiting values of estimators arising from CIF-based FG and DB analyses. If $\kappa_1 = \kappa_2 = 1$, then $\lambda_{0k}(t|X) = \lambda_k \exp(\gamma_k X)$ and we retrieve the time-homogeneous setting of Section 3.2 and Section S1.1. For given values of $\kappa_1 > 0$ and $\kappa_2 > 0$, we chose λ_1 and λ_2 such that $P(T \leq 1 | X = 0) = 0.6$ and $P(T_1 < T_2 | T \leq 1, X = 0) = 0.4, 0.6$ or 0.8. For illustration, Figure S5 contains plots of $\lambda_{01}(t|X = 0)$, $\lambda_{02}(t|X = 0)$ and $F_1(t|X = 0)$ when $\kappa_1 = 0.8, 1, 1.2, 1.4$ and $\kappa_2 = 1$. When $\kappa_1 > 1$ (or < 1), the $0-1$ intensity is increasing (or decreasing) over time; see left panel of Figure S5.
We next assessed the adequacy of the cloglog model (3.1) by calculating $g(F_1(t|X = 1)) - g(F_1(t|X = 0))$ based on the true values of $F_1(t|X)$ (see (2.4)) for $t \in (0, 1)$ and $g(u) = \log(-\log(1 - u))$; results are displayed in Figure S6. We note that departures from a horizontal line are indicative of model misspecification. For a fixed $\exp(\gamma_2)$, the transformation model improves with decreasing

 © xx Copyright Holder Name

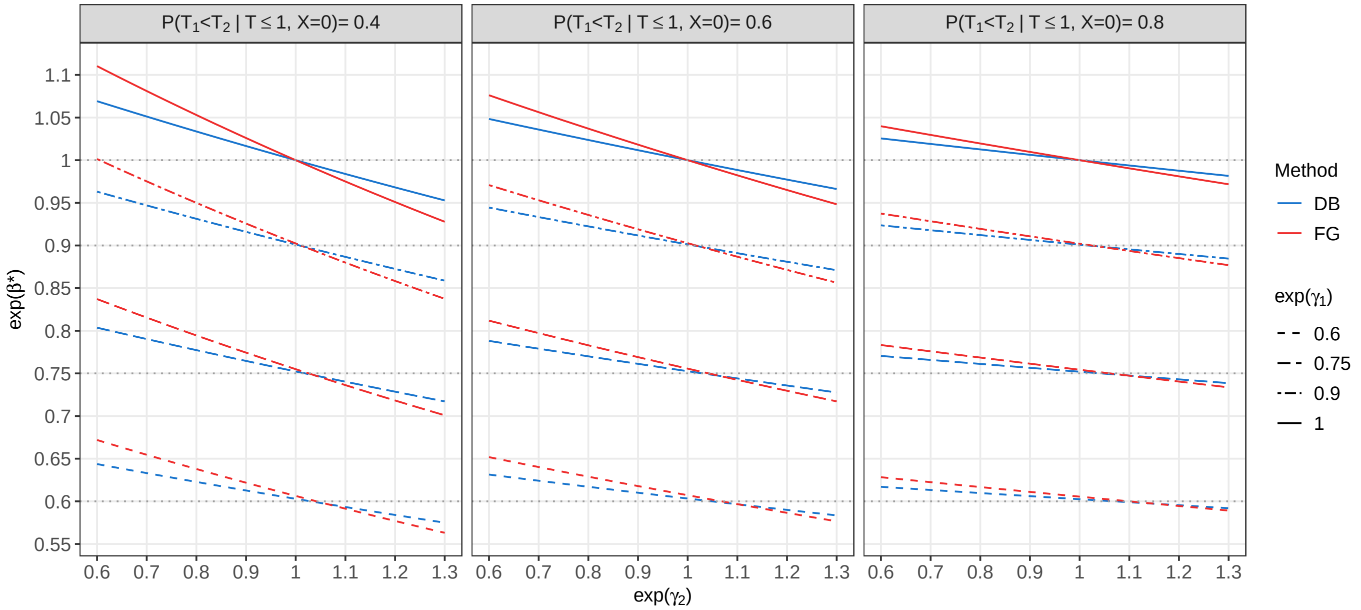

FIGURE S1 Limiting values $\exp(\beta^\star_{FG})$ and $\exp(\beta^\star_{DB})$ as a function of $\exp(\gamma_2)$ for different values of $\exp(\gamma_1)$ and $P(T_1 < T_2|T \leq 1, X = 0)$ when the true process has intensities $\lambda_{0k}(t|X) = \lambda_k \exp(\gamma_k X)$; $\tau = 1, P(T \leq 1|X = 0) = 0.6$, DB estimation is based on $R = 6$ equi-spaced time points in $(0, \tau)$, $G^\star = G$.

κ_1 and is, thus, a better approximation when $\kappa_1 = 1$ as compared to when $\kappa_1 > 1$; the approximation is even better when the intensities in the true process are decreasing over time (i.e. $\kappa_1 < 1$).
We then derived the limiting values $\beta^\star_{FG}$ by solving (3.9) and $\beta^\star_{DB}$ by solving (3.15)-(3.16), respectively. Figures S7 and S8 display plots of $\exp(\beta^\star_{FG})$ and $\exp(\beta^\star_{DB})$ as a function of κ_1 when $\kappa_2 = 1$ and the adopted censoring model is correctly specified; this dependency is illustrated for different values of $\exp(\gamma_1), \exp(\gamma_2)$ and the proportion of type 1 events by the administrative censoring time $\tau = 1$ in the control group (here through $P(T_1 < T_2|T \leq 1, X = 0)$). Figures S7(a) and S8(a) show that $\beta^\star_{FG}$ and $\beta^\star_{DB}$ are unaffected by κ_1 if $\exp(\gamma_1) = \exp(\gamma_2) = 1$; see black lines. For all other scenarios, the dependency of $\beta^\star_{FG}$ and $\beta^\star_{DB}$ on κ_1 becomes stronger as the magnitude of γ_2 increases and as $P(T_1 < T_2|T \leq 1, X = 0)$ decreases. Interestingly, the DB approach seems more sensitive to time-varying intensities than the FG approach when $\exp(\gamma_1) = 1$, but less sensitive when $\exp(\gamma_1) = 0.75$.

S2 | ADDITIONAL RESULTS FOR THE SIMULATION STUDY OF SECTION 4.2

In this section we present additional simulation results; the simulation setup has been described in Section 4.2.1. Tables S1 and S2 report the empirical rejection rates for various tests for the setting where the true process is governed by intensity functions of the form $\lambda_{0k}(t|X) = \lambda_k \exp(\gamma_k X), k = 1, 2$ and where $P(T_1 < T_2|T \leq 1, X = 0) = 0.4$ or 0.8, along with the corresponding limiting values $\beta^\star_{FG}, \beta^\star_{DB_6}$ and $\beta^\star_{DB_3}$ under FG and DB estimation. Accordingly, Table S3 represents the corresponding empirical rejection rates when data were generated according to models (4.7)-(4.8) and (4.9)-(4.10), and when $F_1(1|X = 0) = 0.24$ and 0.48.
The simulation results again confirm that $T^{F_1}_{FG}, T^{F_1}_{DB_6}$ and $T^{F_1}_{DB_3}$ are valid Wald tests of $\beta = 0$ under $H_0^{\lambda_1\lambda_2}$ and H_0, provided robust variance estimates are used. If the true data-generating process is implied by $H_0^{\lambda_1}$, these tests are no longer valid in the sense that they do not control the nominal 5% level. This is particularly the case when the treatment effect γ_2 on the $0 - 2$ intensity is reasonably large. If $\exp(\gamma_2)$ is close to 1, the type I error rate inflation is negligible. When comparing results presented in Tables S1 and S2, we see that the magnitude of inflation is also determined by the probability observed events are of type 1 in the control arm.
Simulation results based on trials with sample sizes of $n = 500$ and $n = 250$ are reported in Tables S4 and S7 for the intensity-based data-generating mechanism, and in Tables S5 and S6 for processes implied by models (4.7)-(4.8) and (4.9)-(4.10), respectively. For all scenarios considered, the type I error rate inflation of tests $T^{F_1}_{FG}, T^{F_1}_{DB_6}$ and $T^{F_1}_{DB_3}$ under $H_0^{\lambda_1}$ is at most 0.075 when $n = 500$ and 0.065 when $n = 250$. All tests for H_0 maintain the nominal 5% level under H_0, even for small sample sizes.

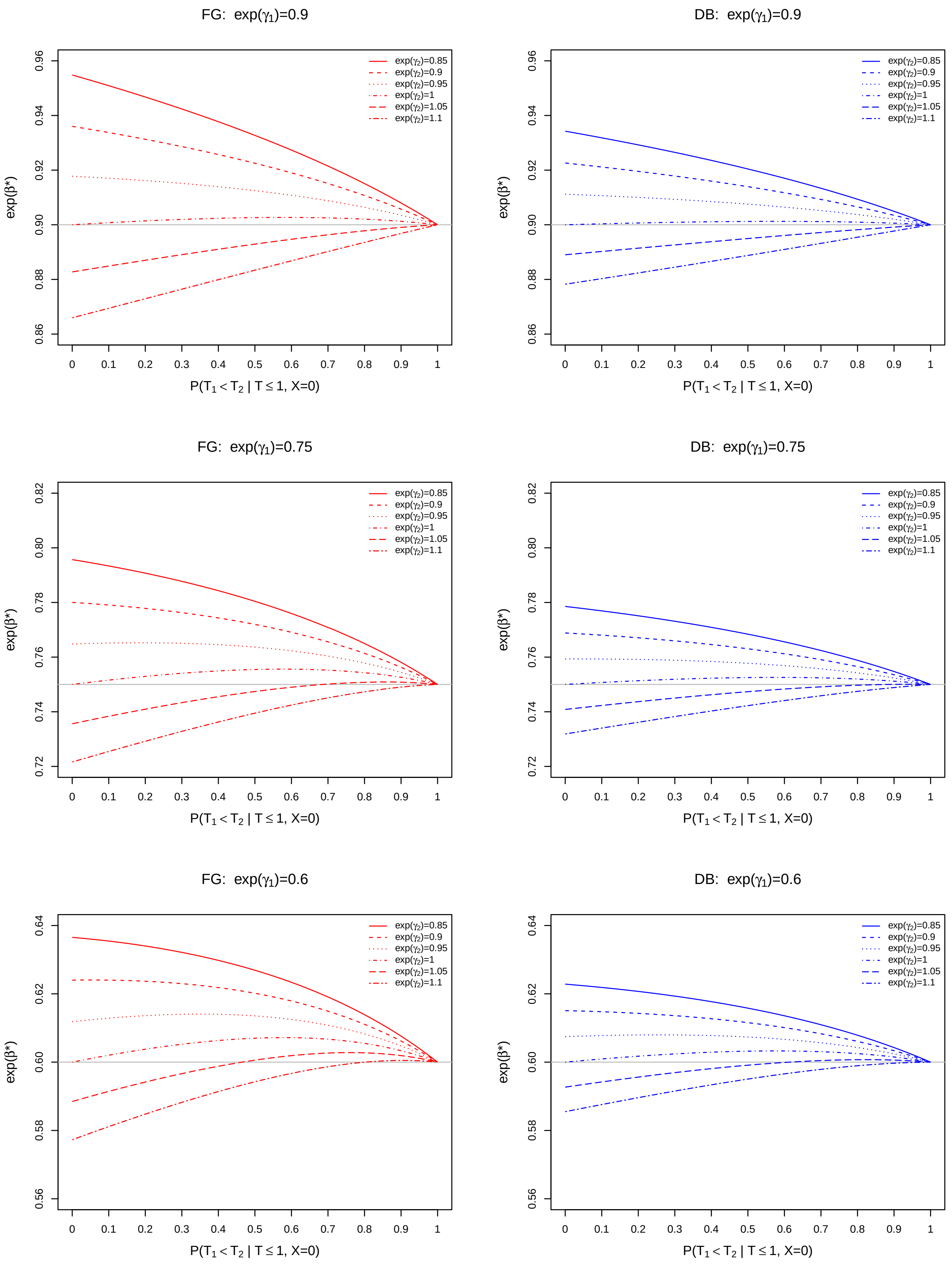

FIGURE S2 Limiting values $\exp(\beta^{\star}_{FG})$ and $\exp(\beta^{\star}_{DB})$ as a function of $P(T_1 < T_2 | T \leq 1, X = 0)$ for different values of $\exp(\gamma_2)$ and $\exp(\gamma_1)$ when the true process has intensities $\lambda_{0k}(t|X) = \lambda_k \exp(\gamma_k X)$; $\tau = 1, P(T \leq 1|X = 0) = 0.6$, DB estimation is based on $R = 6$ equi-spaced time points in $(0, \tau)$, $G^{\star} = G$.

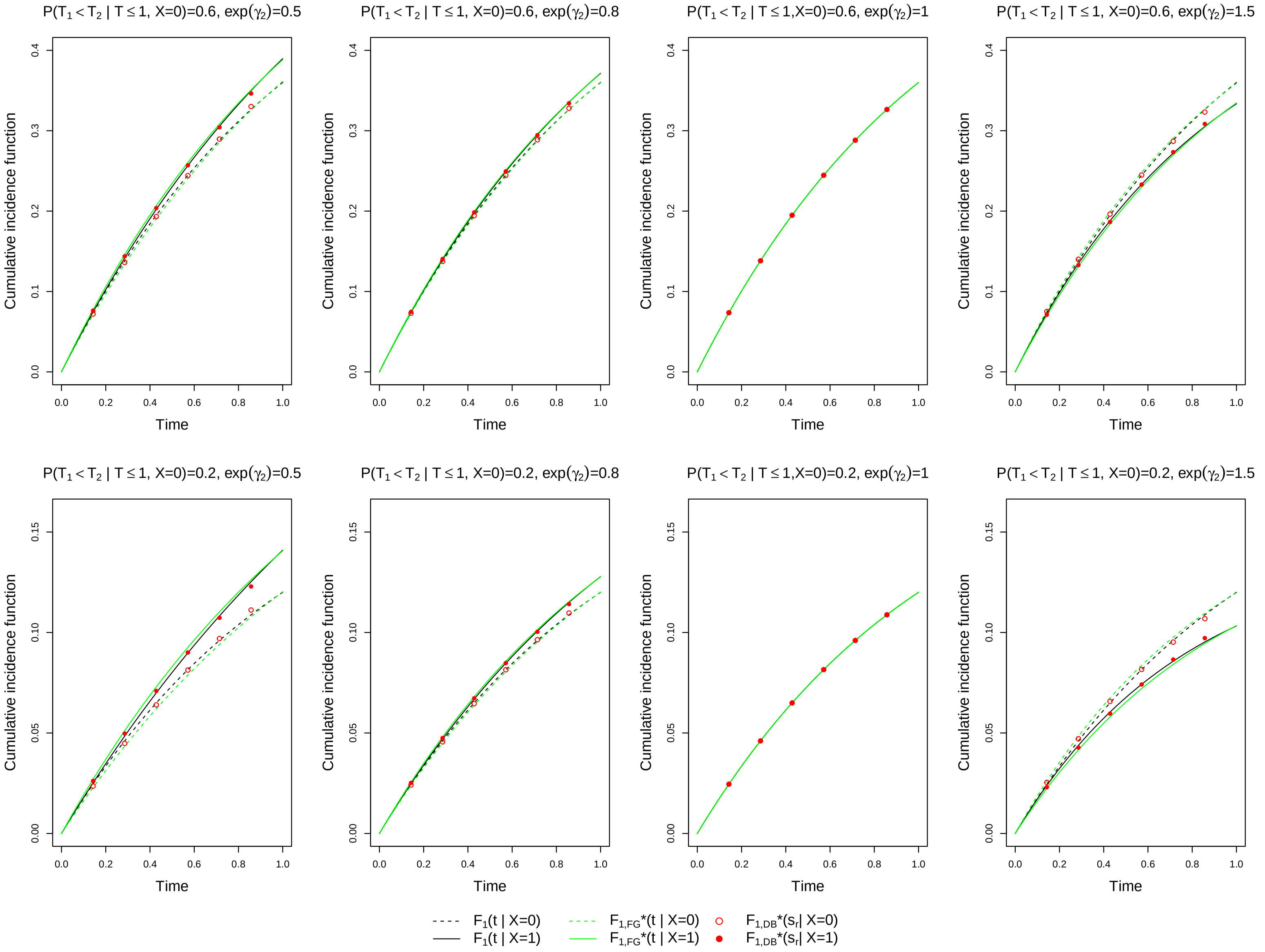

FIGURE S3 Comparisons of $F_1(t|X) = \int_0^t \lambda_{01}(u|X)S(u^-|X)du$, $F^\star_{1,FG}(t|X) = h(\alpha^\star_{FG}(t) + \beta^\star_{FG}X)$ and $F^\star_{1,DB}(s_r|X) = h(\alpha^\star_{rDB} + \beta^\star_{DB}X)$, $X = 0, 1$ when the true process has intensities $\lambda_{0k}(t|X) = \lambda_k \exp(\gamma_k X)$ for $\exp(\gamma_1) = 1$, $\exp(\gamma_2) = 0.5, 0.8, 1, 1.5$ and $P(T_1 < T_2 | T \leq 1, X = 0) = 0.6$ (top row) and 0.2 (bottom row); $\tau = 1$, $P(T \leq 1 | X = 0) = 0.6$, DB procedure is based on $R = 6$ equi-spaced time points in $(0, \tau)$, $G^\star = G$.

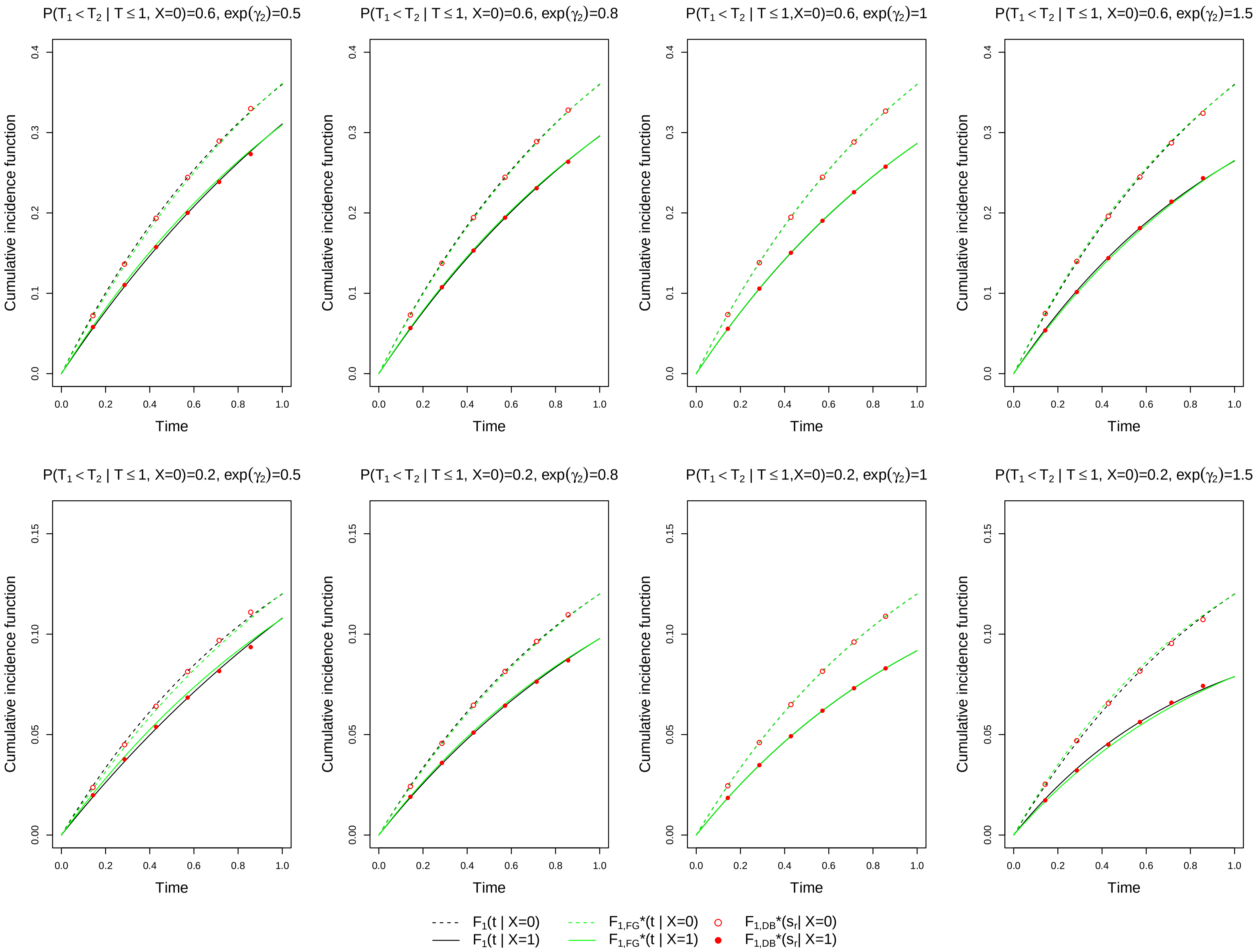

FIGURE S4 Comparisons of $F_1(t|X) = \int_0^t \lambda_{01}(u|X)S(u^-|X)du$, $F^\star_{1,FG}(t|X) = h(\alpha^\star_{FG}(t) + \beta^\star_{FG}X)$ and $F^\star_{1,DB}(s_r|X) = h(\alpha^\star_{rDB} + \beta^\star_{DB}X)$, $X = 0, 1$ when the true process has intensities $\lambda_{0k}(t|X) = \lambda_k \exp(\gamma_k X)$ for $\exp(\gamma_1) = 0.75$, $\exp(\gamma_2) = 0.5, 0.8, 1, 1.5$ and $P(T_1 < T_2|T \leq 1, X = 0) = 0.6$ (top row) and 0.2 (bottom row); $\tau = 1$, $P(T \leq 1|X = 0) = 0.6$, DB procedure is based on $R = 6$ equi-spaced time points in $(0, \tau)$, $G^\star = G$.

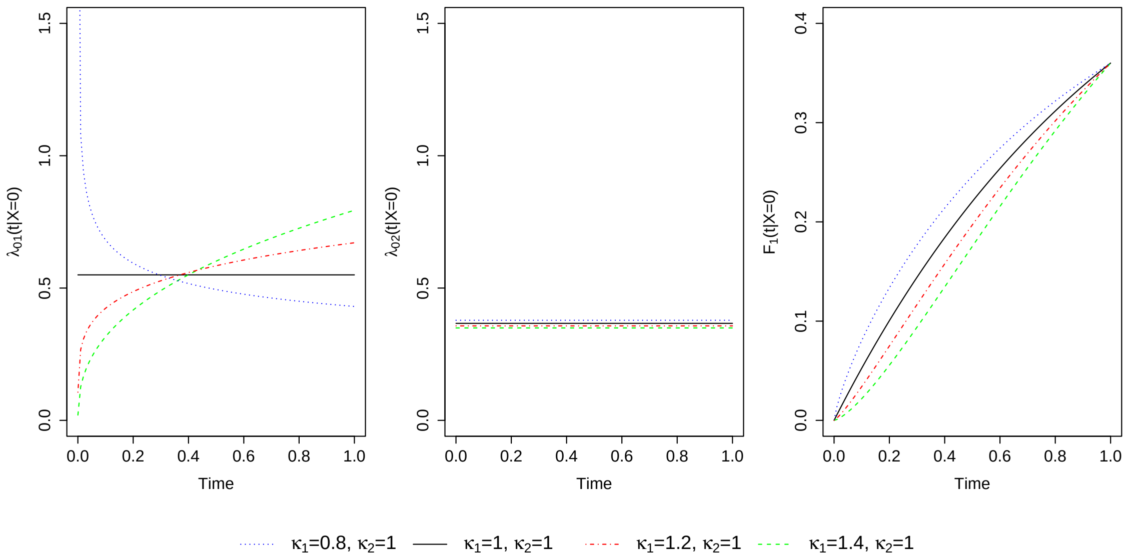

FIGURE S5 Plots of $\lambda_{01}(t|X = 0)$, $\lambda_{02}(t|X = 0)$ and $F_1(t|X = 0)$ for different values of κ_1 when the true competing risks process has baseline intensities of Weibull form $\lambda_k(t) = \kappa_k\lambda_k(\lambda_k t)^{\kappa_k - 1}$; $\kappa_2 = 1, \tau = 1, P(T \leq 1|X = 0) = 0.6, P(T_1 < T_2|T \leq 1, X = 0) = 0.6$.

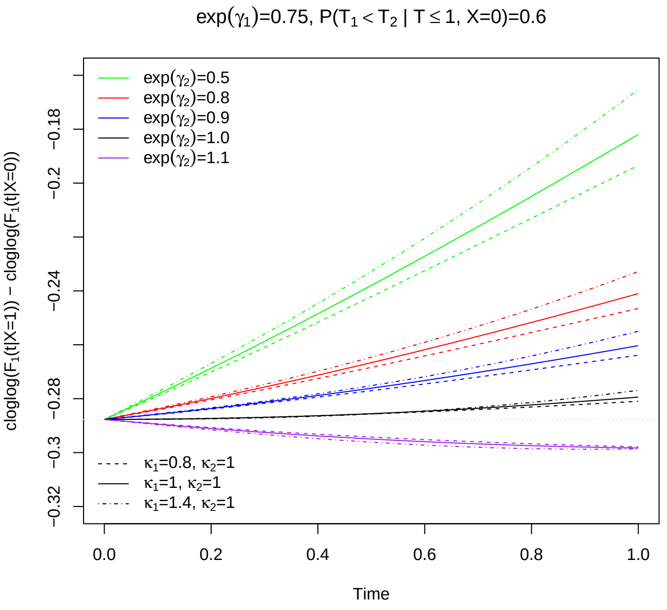

FIGURE S6 Plots of $g(F_1(t|X = 1)) - g(F_1(t|X = 0))$ for $t \in (0, 1)$, $g(u) = \log(-\log(1 - u))$ and different values of κ_1 and $\exp(\gamma_2)$, $\kappa_2 = 1, \tau = 1, P(T \leq 1|X = 0) = 0.6, \gamma_1 = \log(0.75) = -0.288$.

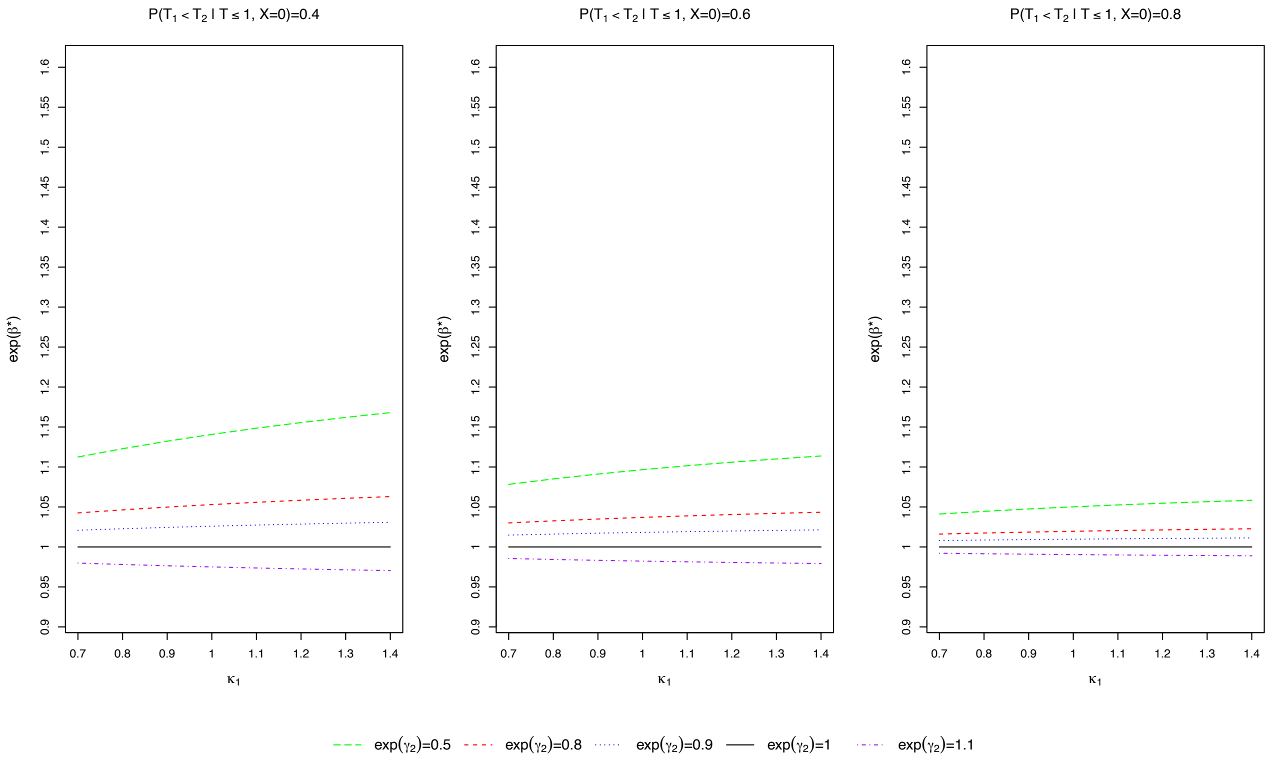

(a) $\exp(\gamma_1) = 1, \kappa_2 = 1$.

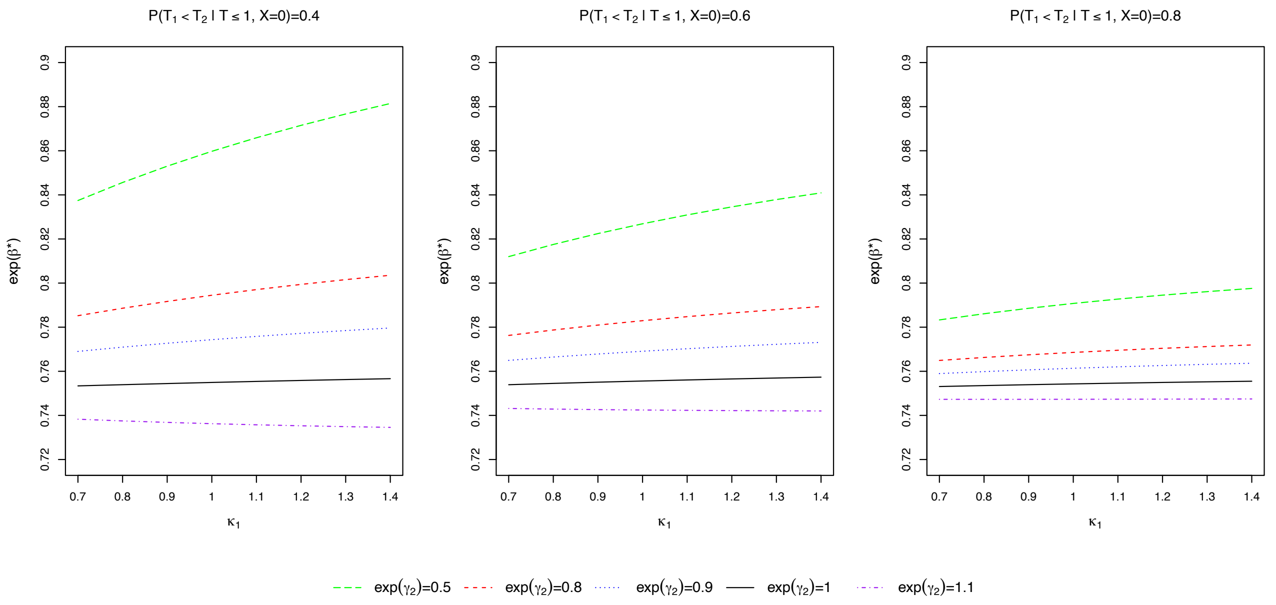

(b) $\exp(\gamma_1) = 0.75, \kappa_2 = 1$.

FIGURE S7 Limiting values $\exp(\beta^{\star}_{FG})$ of the FG estimator as a function of κ_1 for different values of $\exp(\gamma_2)$ and $P(T_1 < T_2 | T \leq 1, X = 0)$ when the true process has Weibull intensities $\lambda_{0k}(t|X) = \kappa_k \lambda_k (\lambda_k t)^{\kappa_k - 1} \exp(\gamma_k X)$ with $\exp(\gamma_1) = 1$ (top row) and $e^{\gamma_1} = 0.75$ (bottom row); $\kappa_2 = 1, \tau = 1, P(T \leq 1 | X = 0) = 0.6, G^{\star} = G$.

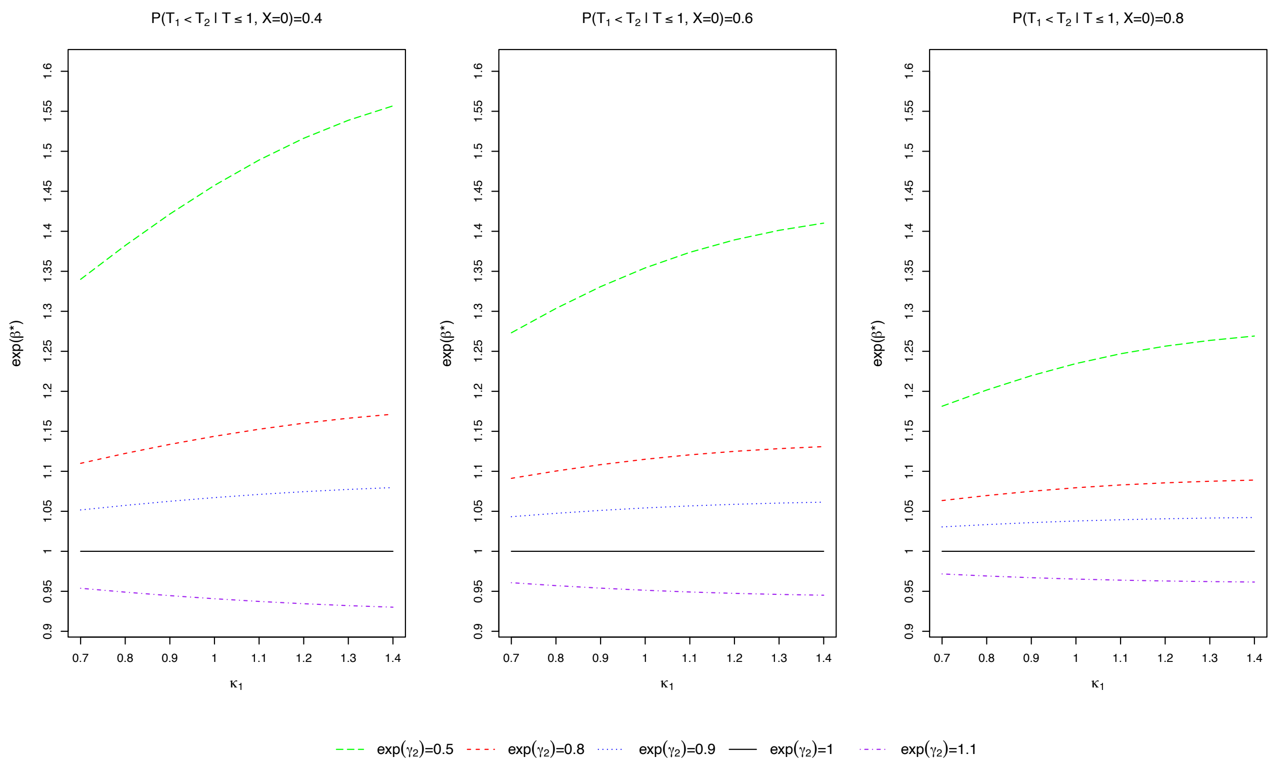

(a) $\exp(\gamma_1) = 1, \kappa_2 = 1$.

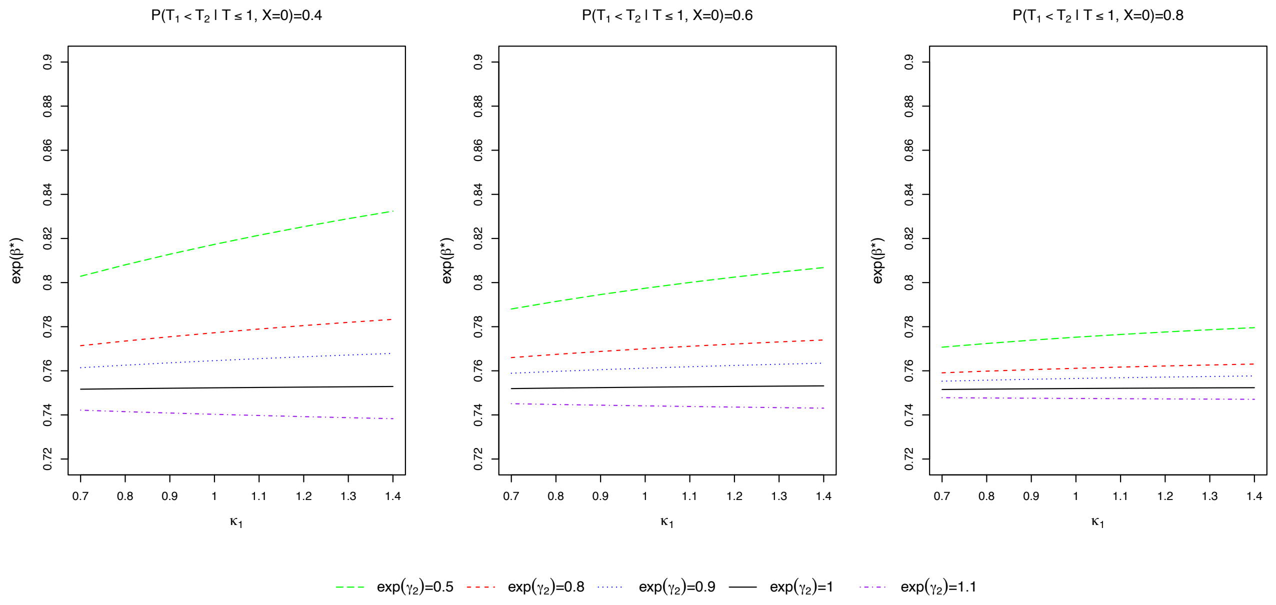

(b) $\exp(\gamma_1) = 0.75, \kappa_2 = 1$.

FIGURE S8 Limiting values $\exp(\beta^\star_{DB})$ of the DB estimator as a function of κ_1 for different values of $\exp(\gamma_2)$ and $P(T_1 < T_2|T \leq 1, X = 0)$ when the true process has Weibull intensities $\lambda_{0k}(t|X) = \kappa_k \lambda_k (\lambda_k t)^{\kappa_k - 1} \exp(\gamma_k X)$ for $\exp(\gamma_1) = 1$ (top row) and $\exp(\gamma_1) = 0.75$ (bottom row); $\kappa_2 = 1, R = 6, \tau = 1, P(T \leq 1|X = 0) = 0.6, G^\star = G$.

		REJECTION RATES							PROBABILITY LIMITS		
		$H_0^{\lambda_1}$		$H_0^{\lambda_1\lambda_2}$	H_0						
$\exp(\gamma_2)$	$\exp(\gamma_1)$	$T_{LR}^{\lambda_1}$	$T_{Cox}^{\lambda_1}$	$T_{Cox}^{\lambda_1\lambda_2}$	$T_{Gray}^{F_1}$	$T_{FG}^{F_1}$	$T_{DB_6}^{F_1}$	$T_{DB_3}^{F_1}$	$\beta_{FG}^{\star}$	$\beta_{DB_6}^{\star}$	$\beta_{DB_3}^{\star}$
0.5	1	0.0497	0.0487	0.9965	0.1324	0.1315	0.0901	0.0844	0.1183	0.0841	0.0797
	0.9	0.1154	0.1138	0.9986	0.0520	0.0515	0.0530	0.0542	0.0147	-0.0205	-0.0250
	0.75	0.4940	0.4910	0.9996	0.2003	0.2000	0.2291	0.2267	-0.1649	-0.2017	-0.2064
	0.6	0.9088	0.9072	1.0000	0.6973	0.6956	0.6946	0.6829	-0.3853	-0.4237	-0.4285
0.8	1	0.0517	0.0513	0.3423	0.0626	0.0624	0.0605	0.0574	0.0463	0.0331	0.0314
	0.9	0.1147	0.1133	0.4025	0.0693	0.0688	0.0742	0.0736	-0.057	-0.0712	-0.0730
	0.75	0.4749	0.4717	0.6471	0.3506	0.3482	0.3370	0.3267	-0.2362	-0.2520	-0.2540
	0.6	0.8941	0.8923	0.9216	0.8142	0.8122	0.7778	0.7621	-0.4562	-0.4736	-0.4757
0.9	1	0.0484	0.0477	0.1076	0.0494	0.0487	0.0485	0.0473	0.0230	0.0165	0.0156
	0.9	0.1129	0.1116	0.1603	0.0869	0.0862	0.0858	0.0842	-0.0802	-0.0878	-0.0887
	0.75	0.4709	0.4684	0.4477	0.3994	0.3954	0.3657	0.3508	-0.2593	-0.2684	-0.2695
	0.6	0.8839	0.8826	0.8423	0.8437	0.8440	0.8016	0.7866	-0.4792	-0.4899	-0.4912
1	1	0.0523	0.0508	0.0443	0.0516	0.0509	0.0484	0.0492	0.0000	0.0000	0.0000
	0.9	0.1108	0.1094	0.0883	0.1084	0.1078	0.1030	0.0987	-0.1031	-0.1041	-0.1043
	0.75	0.4652	0.4626	0.3640	0.4553	0.4526	0.4070	0.3879	-0.2821	-0.2846	-0.2850
	0.6	0.8890	0.8881	0.8154	0.8792	0.8776	0.8373	0.8193	-0.5019	-0.5060	-0.5065
1.1	1	0.0533	0.0525	0.1021	0.0561	0.0566	0.0547	0.0542	-0.0226	-0.0163	-0.0155
	0.9	0.1027	0.1019	0.1417	0.1266	0.1267	0.1108	0.1083	-0.1257	-0.1203	-0.1197
	0.75	0.4659	0.4635	0.4316	0.5115	0.5083	0.4461	0.4275	-0.3046	-0.3007	-0.3003
	0.6	0.8781	0.8768	0.8322	0.8917	0.8908	0.8473	0.8298	-0.5243	-0.5220	-0.5217
1.5	1	0.0435	0.0424	0.9227	0.1129	0.1112	0.0847	0.0781	-0.1100	-0.0797	-0.0760
	0.9	0.1099	0.1090	0.9340	0.2949	0.2933	0.2257	0.2090	-0.2129	-0.1835	-0.1799
	0.75	0.4411	0.4388	0.9684	0.6978	0.6951	0.5952	0.5584	-0.3915	-0.3635	-0.3601
	0.6	0.8654	0.8635	0.9940	0.9565	0.9551	0.9130	0.8962	-0.6110	-0.5844	-0.5812

TABLE S1 Empirical rejection rates for different statistical tests of nominal size $\omega = 0.05$ when the true process has intensities $\lambda_{0k}(t|X) = \lambda_k \exp(\gamma_k X)$, along with the limiting values $\beta_{FG}^{\star}$ and $\beta_{DB}^{\star}$; γ_1 : $\log(0.9) = -0.1054, \log(0.75) = -0.2877, \log(0.6) = -0.5108$; $n = 1000$ individuals, $n_{sim} = 10000$, $\tau = 1$, $P(T \leq 1|X = 0) = 0.6, P(T_1 < T_2|T \leq 1, X = 0) = 0.4, \pi_r = 0.2$.

		REJECTION RATES							PROBABILITY LIMITS		
		$H_0^{\lambda_1}$		$H_0^{\lambda_1\lambda_2}$	H_0						
$\exp(\gamma_2)$	$\exp(\gamma_1)$	$T_{LR}^{\lambda_1}$	$T_{Cox}^{\lambda_1}$	$T_{Cox}^{\lambda_1\lambda_2}$	$T_{Gray}^{F_1}$	$T_{FG}^{F_1}$	$T_{DB_6}^{F_1}$	$T_{DB_3}^{F_1}$	$\beta_{FG}^{\star}$	$\beta_{DB_6}^{\star}$	$\beta_{DB_3}^{\star}$
0.5	1	0.0517	0.0512	0.7181	0.0748	0.0739	0.0592	0.0585	0.0436	0.0316	0.0297
	0.9	0.1730	0.1718	0.7887	0.0913	0.0911	0.0968	0.0977	-0.0604	-0.0732	-0.0751
	0.75	0.7710	0.7697	0.9562	0.6120	0.6115	0.5777	0.5618	-0.2406	-0.2546	-0.2567
	0.6	0.9963	0.9963	0.9994	0.9861	0.9862	0.9757	0.9719	-0.4615	-0.4768	-0.4791
0.8	1	0.0520	0.0516	0.1320	0.0552	0.0542	0.0548	0.0556	0.0172	0.0125	0.0118
	0.9	0.1794	0.1783	0.2355	0.1356	0.1363	0.1313	0.1266	-0.0864	-0.0919	-0.0928
	0.75	0.7704	0.7688	0.7358	0.7013	0.7003	0.6424	0.6227	-0.2661	-0.2729	-0.2740
	0.6	0.9964	0.9963	0.9923	0.9931	0.9930	0.9848	0.9810	-0.4866	-0.4948	-0.4960
0.9	1	0.0502	0.0500	0.0692	0.0515	0.0520	0.0504	0.0498	0.0086	0.0063	0.0059
	0.9	0.1728	0.1722	0.1557	0.1495	0.1501	0.1351	0.1323	-0.0949	-0.0981	-0.0986
	0.75	0.7616	0.7601	0.6789	0.7271	0.7264	0.6629	0.6406	-0.2745	-0.2790	-0.2797
	0.6	0.9949	0.9949	0.9883	0.9934	0.9934	0.9833	0.9788	-0.4948	-0.5007	-0.5016
1	1	0.0499	0.0498	0.0462	0.0500	0.0504	0.0479	0.0508	0.0000	0.0000	0.0000
	0.9	0.1736	0.1727	0.1331	0.1717	0.1703	0.1506	0.1492	-0.1034	-0.1043	-0.1044
	0.75	0.7569	0.7553	0.6555	0.7421	0.7415	0.6738	0.6484	-0.2829	-0.2851	-0.2854
	0.6	0.9950	0.9949	0.9864	0.9945	0.9946	0.9871	0.9842	-0.5031	-0.5067	-0.5072
1.1	1	0.0517	0.0516	0.0685	0.0514	0.0512	0.0488	0.0494	-0.0085	-0.0062	-0.0059
	0.9	0.1701	0.1691	0.1467	0.1885	0.1878	0.1594	0.1549	-0.1119	-0.1104	-0.1102
	0.75	0.7622	0.7610	0.6775	0.7746	0.7739	0.7035	0.6812	-0.2912	-0.2911	-0.2911
	0.6	0.9943	0.9942	0.9869	0.9939	0.9940	0.9879	0.9831	-0.5113	-0.5126	-0.5128
1.5	1	0.0527	0.0526	0.4758	0.0705	0.0703	0.0594	0.0579	-0.0422	-0.0308	-0.0291
	0.9	0.1684	0.1671	0.5613	0.2794	0.2795	0.2194	0.2074	-0.1451	-0.1347	-0.1331
	0.75	0.7578	0.7565	0.8807	0.8476	0.8473	0.7711	0.7447	-0.3240	-0.3149	-0.3135
	0.6	0.9944	0.9944	0.9971	0.9981	0.9979	0.9931	0.9902	-0.5437	-0.5360	-0.5348

TABLE S2 Empirical rejection rates for different statistical tests of nominal size $\omega = 0.05$ when the true process has intensities $\lambda_{0k}(t|X) = \lambda_k \exp(\gamma_k X)$, along with the limiting values $\beta_{FG}^{\star}$ and $\beta_{DB}^{\star}$; γ_1 : $\log(0.9) = -0.1054, \log(0.75) = -0.2877, \log(0.6) = -0.5108$; $n = 1000$ individuals, $n_{sim} = 10000$, $\tau = 1$, $P(T \leq 1|X = 0) = 0.6, P(T_1 < T_2|T \leq 1, X = 0) = 0.8, \pi_r = 0.2$.

				REJECTION RATES								PROBABILITY LIMITS
				$H_0^{\lambda_1}$		$H_0^{\lambda_1\lambda_2}$	$H_0^{F_1}$					
Models	$F_1(1\|X=0)$	$\exp(\beta)$	$\exp(\beta_2)$	$T_{LR}^{\lambda_1}$	$T_{Cox}^{\lambda_1}$	$T_{Cox}^{\lambda_1\lambda_2}$	$T_{Gray}^{F_1}$	$T_{FG}^{F_1}$	$T_{DB_6}^{F_1}$	$T_{DB_3}^{F_1}$		$\beta_{FG}^\star, \beta_{DB_6}^\star, \beta_{DB_3}^\star$
(4.7) - (4.8)	0.24	0.8	-	0.3908	0.3882	0.3878	0.3134	0.3117	0.2816	0.2724		-0.2232
		0.9		0.1247	0.1232	0.1171	0.1076	0.1072	0.0988	0.0975		-0.1054
		1		0.0507	0.0495	0.0527	0.0512	0.0519	0.0539	0.0535		0.0000
		1.1		0.1152	0.1144	0.1035	0.0998	0.0986	0.0932	0.0926		0.0953
	0.48	0.8	-	0.5319	0.5300	0.4480	0.5485	0.5472	0.4872	0.4670		-0.2231
		0.9		0.1704	0.1690	0.1348	0.1793	0.1799	0.1583	0.1544		-0.1054
		1		0.0500	0.0498	0.0479	0.0502	0.0502	0.0470	0.0471		0.0000
		1.1		0.1504	0.1495	0.1252	0.1623	0.1621	0.1439	0.1369		0.0953
(4.9) - (4.10)	0.48	0.8	0.8	0.5501	0.5489	0.4644	0.5636	0.5639	0.4984	0.4794		-0.2231
			1	0.4465	0.4455	0.5584	0.5528	0.5513	0.4883	0.4680		-0.2231
		1	0.8	0.0572	0.0570	0.1134	0.0501	0.0497	0.0499	0.0496		0.0000
			1	0.0484	0.0478	0.0466	0.0484	0.0495	0.0492	0.0481		0.0000

TABLE S3 Empirical rejection rates for different statistical tests of nominal size $\omega = 0.05$ when the true process was implied by models (4.7)-(4.8) and (4.9)-(4.10), along with the limiting values $\beta_{FG}^\star$ and $\beta_{DB}^\star$; β : log(0.8) = –0.2231, log(0.9) = –0.1054, log(1.1) = 0.0953; $n = 1000$ individuals, $n_{sim} = 10000$, $\tau = 1, F_1(1|X = 0) = 0.24$ or 0.48, $F_2(1|X = 0) = 0.39$ or 0.15, $\pi_r = 0.2$.

		REJECTION RATES								PROBABILITY LIMITS		
		$H_0^{\lambda_1}$		$H_0^{\lambda_1\lambda_2}$	H_0							
$\exp(\gamma_2)$	$\exp(\gamma_1)$	$T_{LR}^{\lambda_1}$	$T_{Cox}^{\lambda_1}$	$T_{Cox}^{\lambda_1\lambda_2}$	$T_{Gray}^{F_1}$	$T_{FG}^{F_1}$	$T_{DB_6}^{F_1}$	$T_{DB_3}^{F_1}$	$\beta_{FG}^{\star}$	$\beta_{DB_6}^{\star}$	$\beta_{DB_3}^{\star}$
0.5	1	0.0504	0.0494	0.7312	0.0753	0.0749	0.0622	0.0609	0.0825	0.0593	0.0560
	0.9	0.0991	0.0974	0.7611	0.0527	0.0516	0.0575	0.0589	-0.0210	-0.0453	-0.0487
	0.75	0.3814	0.3784	0.8700	0.2136	0.2141	0.2210	0.2135	-0.2003	-0.2264	-0.2299
	0.6	0.7986	0.7951	0.9629	0.6375	0.6369	0.6141	0.5990	-0.4205	-0.4483	-0.4520
0.8	1	0.0503	0.0494	0.1350	0.0554	0.0545	0.0511	0.0527	0.0324	0.0234	0.0221
	0.9	0.0933	0.0921	0.1701	0.0683	0.0682	0.0691	0.0713	-0.0706	-0.0808	-0.0822
	0.75	0.3788	0.3744	0.3982	0.2957	0.2944	0.2777	0.2690	-0.2495	-0.2614	-0.2630
	0.6	0.7967	0.7935	0.7631	0.7274	0.7256	0.6765	0.6579	-0.4691	-0.4828	-0.4846
0.9	1	0.0508	0.0501	0.0680	0.0519	0.0514	0.0509	0.0510	0.0161	0.0117	0.0110
	0.9	0.0959	0.0941	0.0966	0.0804	0.0805	0.0766	0.0751	-0.0868	-0.0924	-0.0932
	0.75	0.3702	0.3671	0.3071	0.3259	0.3246	0.2915	0.2791	-0.2655	-0.2728	-0.2738
	0.6	0.7889	0.7856	0.7090	0.7490	0.7478	0.6936	0.6709	-0.4850	-0.4941	-0.4953
1	1	0.0477	0.0466	0.0478	0.0476	0.0471	0.0496	0.0519	0.0000	0.0000	0.0000
	0.9	0.0979	0.0962	0.0732	0.0946	0.0937	0.0889	0.0847	-0.1028	-0.1040	-0.1041
	0.75	0.3684	0.3648	0.2811	0.3577	0.3563	0.3141	0.3003	-0.2814	-0.2842	-0.2846
	0.6	0.7816	0.7785	0.6894	0.7655	0.7635	0.7112	0.6873	-0.5007	-0.5054	-0.5060
1.1	1	0.0512	0.0503	0.0628	0.0538	0.0532	0.0523	0.0525	-0.0159	-0.0116	-0.0109
	0.9	0.0925	0.0906	0.0947	0.1040	0.1039	0.0955	0.0923	-0.1187	-0.1154	-0.1150
	0.75	0.3707	0.3666	0.2991	0.3899	0.3881	0.3387	0.3258	-0.2971	-0.2956	-0.2954
	0.6	0.7847	0.7813	0.6948	0.7949	0.7926	0.7306	0.7066	-0.5163	-0.5166	-0.5166
1.5	1	0.0500	0.0489	0.4593	0.0759	0.0751	0.0649	0.0612	-0.0780	-0.0569	-0.0540
	0.9	0.0924	0.0906	0.5091	0.1839	0.1816	0.1440	0.1335	-0.1805	-0.1604	-0.1577
	0.75	0.3570	0.3538	0.6718	0.5136	0.5106	0.4314	0.4056	-0.3585	-0.3400	-0.3376
	0.6	0.7657	0.7625	0.8819	0.8613	0.8593	0.7931	0.7672	-0.5774	-0.5605	-0.5583

TABLE S4 Empirical rejection rates for different statistical tests of nominal size $\omega = 0.05$ when the true process has intensities $\lambda_{0k}(t|X) = \lambda_k \exp(\gamma_k X)$, along with the limiting values $\beta_{FG}^{\star}$ and $\beta_{DB}^{\star}$; γ_1 : log(0.9) = –0.1054, log(0.75) = –0.2877, log(0.6) = –0.5108; $n = 500$ individuals, $n_{sim} = 10000$, $\tau = 1$, $P(T \leq 1|X = 0) = 0.6, P(T_1 < T_2|T \leq 1, X = 0) = 0.6, \pi_r = 0.2$.

				REJECTION RATES							PROBABILITY LIMITS
				$H_0^{\lambda_1}$		$H_0^{\lambda_1\lambda_2}$	$H_0^{F_1}$				
n	Models	$\exp(\beta)$	$\exp(\beta_2)$	$T_{LR}^{\lambda_1}$	$T_{Cox}^{\lambda_1}$	$T_{Cox}^{\lambda_1\lambda_2}$	$T_{Gray}^{F_1}$	$T_{FG}^{F_1}$	$T_{DB_6}^{F_1}$	$T_{DB_3}^{F_1}$	$\beta_{FG}^{\star}, \beta_{DB_6}^{\star}, \beta_{DB_3}^{\star}$
500	(4.7) - (4.8)	0.8	-	0.2668	0.2633	0.1980	0.2467	0.2453	0.2187	0.2092	-0.2231
		0.9		0.0985	0.0972	0.0802	0.0955	0.0942	0.0872	0.0853	-0.1054
		1		0.0498	0.0487	0.0458	0.0511	0.0511	0.0498	0.0502	0.0000
		1.1		0.090	0.0882	0.0758	0.0873	0.0865	0.0800	0.0796	0.0953
	(4.9) - (4.10)	0.8	0.8	0.2706	0.2669	0.2004	0.2451	0.2441	0.2154	0.2062	-0.2231
			1	0.2011	0.1979	0.2176	0.2522	0.2506	0.2262	0.2123	
		0.9	0.8	0.1193	0.1168	0.1198	0.0984	0.0973	0.0911	0.0899	-0.1054
			1	0.0808	0.0800	0.0798	0.0914	0.0906	0.0835	0.0818	
		1	0.8	0.0561	0.0551	0.1203	0.0536	0.0538	0.0538	0.0523	0.0000
			1	0.0513	0.0501	0.0518	0.0500	0.0494	0.0500	0.0502	
		1.1	0.8	0.0662	0.0647	0.1957	0.0959	0.0955	0.0899	0.0857	0.0953
			1	0.0817	0.0804	0.0819	0.0887	0.0885	0.0842	0.0822	

TABLE S5 Empirical rejection rates for different statistical tests of nominal size $\omega = 0.05$ when the true process is implied by models (4.7)-(4.8) and (4.9)-(4.10), along with the limiting values $\beta_{FG}^{\star}$ and $\beta_{DB}^{\star}$; β : log(0.8) = –0.2231, log(0.9) = –0.1054, log(1.1) = 0.0953; $n = 500$ individuals, $n_{sim} = 10000$, $\tau = 1$, $F_1(1|X = 0) = 0.36$, $F_2(1|X = 0) = 0.27$, $\pi_r = 0.2$.

n	Models	$\exp(\beta)$	$\exp(\beta_2)$	REJECTION RATES $H_0^{\lambda_1}$ $T_{LR}^{\lambda_1}$	$T_{Cox}^{\lambda_1}$	$H_0^{\lambda_1\lambda_2}$ $T_{Cox}^{\lambda_1\lambda_2}$	$H_0^{F_1}$ $T_{Gray}^{F_1}$	$T_{FG}^{F_1}$	$T_{DB_6}^{F_1}$	$T_{DB_3}^{F_1}$	PROBABILITY LIMITS $\beta_{FG}^\star, \beta_{DB_6}^\star, \beta_{DB_3}^\star$
250	(4.7) - (4.8)	0.8	-	0.1573	0.1533	0.1191	0.1468	0.1448	0.1310	0.1244	-0.2231
		0.9		0.0687	0.0661	0.0584	0.0692	0.0681	0.0664	0.0633	-0.1054
		1		0.0532	0.0506	0.0489	0.0529	0.0522	0.0507	0.0510	0.0000
		1.1		0.0699	0.0673	0.0598	0.0679	0.0678	0.0649	0.0624	0.0953
	(4.9) - (4.10)	0.8	0.8	0.1608	0.1560	0.1196	0.1500	0.1484	0.1338	0.1310	-0.2231
			1	0.1264	0.1225	0.1219	0.1487	0.1473	0.1322	0.1250	
		0.9	0.8	0.0818	0.0777	0.0704	0.0714	0.0707	0.0639	0.0605	-0.1054
			1	0.0639	0.0622	0.0604	0.0709	0.0687	0.0656	0.0648	
		1	0.8	0.0527	0.0510	0.0759	0.0521	0.0513	0.0491	0.0495	0.0000
			1	0.0489	0.0473	0.0465	0.0477	0.0470	0.0482	0.0469	
		1.1	0.8	0.0552	0.0526	0.1106	0.0705	0.0696	0.0676	0.0674	0.0953
			1	0.0656	0.0628	0.0649	0.0695	0.0691	0.0675	0.0669	

TABLE S6 Empirical rejection rates for different statistical tests of nominal size $\omega = 0.05$ when the true process is implied by models (4.7)-(4.8) and (4.9)-(4.10), along with the limiting values $\beta_{FG}^\star$ and $\beta_{DB}^\star$; β : log(0.8) = –0.2231, log(0.9) = –0.1054, log(1.1) = 0.0953; $n = 250$ individuals, $n_{sim} = 10000$, $\tau = 1$, $F_1(1|X = 0) = 0.36$, $F_2(1|X = 0) = 0.27$, $\pi_r = 0.2$.

		REJECTION RATES							PROBABILITY LIMITS		
		$H_0^{\lambda_1}$		$H_0^{\lambda_1\lambda_2}$	H_0						
$\exp(\gamma_2)$	$\exp(\gamma_1)$	$T_{LR}^{\lambda_1}$	$T_{Cox}^{\lambda_1}$	$T_{Cox}^{\lambda_1\lambda_2}$	$T_{Gray}^{F_1}$	$T_{FG}^{F_1}$	$T_{DB_6}^{F_1}$	$T_{DB_3}^{F_1}$	$\beta_{FG}^{\star}$	$\beta_{DB_6}^{\star}$	$\beta_{DB_3}^{\star}$
0.5	1	0.0526	0.0504	0.4126	0.0661	0.0645	0.0588	0.0587	0.0825	0.0593	0.0560
	0.9	0.0703	0.0678	0.4337	0.0472	0.0465	0.0511	0.0517	-0.0210	-0.0453	-0.0487
	0.75	0.2188	0.2114	0.5442	0.1278	0.1256	0.1341	0.1307	-0.2003	-0.2264	-0.2299
	0.6	0.5123	0.5037	0.7268	0.3709	0.3665	0.3514	0.3376	-0.4205	-0.4483	-0.4520
0.8	1	0.0451	0.0431	0.0841	0.0478	0.0461	0.0470	0.0453	0.0324	0.0234	0.0221
	0.9	0.0762	0.0724	0.1046	0.0596	0.0586	0.0596	0.0580	-0.0706	-0.0808	-0.0822
	0.75	0.2063	0.2010	0.2018	0.1657	0.1644	0.1533	0.1495	-0.2495	-0.2614	-0.2630
	0.6	0.4993	0.4912	0.4396	0.4328	0.4301	0.3927	0.3786	-0.4691	-0.4828	-0.4846
0.9	1	0.0498	0.0467	0.0566	0.0523	0.0518	0.0481	0.0509	0.0161	0.0117	0.0110
	0.9	0.0693	0.0672	0.0685	0.0642	0.0624	0.0615	0.0587	-0.0868	-0.0924	-0.0932
	0.75	0.2120	0.2055	0.1685	0.1839	0.1826	0.1703	0.1615	-0.2655	-0.2728	-0.2738
	0.6	0.4873	0.4781	0.3871	0.4509	0.4469	0.4077	0.3907	-0.4850	-0.4941	-0.4953
1	1	0.0501	0.0482	0.0456	0.0520	0.0519	0.0526	0.0523	0.0000	0.0000	0.0000
	0.9	0.0728	0.0705	0.0591	0.0705	0.0694	0.0680	0.0649	-0.1028	-0.1040	-0.1041
	0.75	0.2136	0.2085	0.1556	0.2073	0.2043	0.1814	0.1736	-0.2814	-0.2842	-0.2846
	0.6	0.4977	0.4888	0.3874	0.4828	0.4791	0.4303	0.4084	-0.5007	-0.5054	-0.5060
1.1	1	0.0508	0.0484	0.0539	0.0508	0.0496	0.0529	0.0500	-0.0159	-0.0116	-0.0109
	0.9	0.0702	0.0669	0.0672	0.0817	0.0811	0.0742	0.0744	-0.1187	-0.1154	-0.1150
	0.75	0.2063	0.2010	0.1618	0.2134	0.2110	0.1870	0.1805	-0.2971	-0.2956	-0.2954
	0.6	0.4984	0.4886	0.3919	0.5080	0.5040	0.4468	0.4246	-0.5163	-0.5166	-0.5166
1.5	1	0.0519	0.0502	0.2415	0.0623	0.0614	0.0530	0.0520	-0.0780	-0.0569	-0.0540
	0.9	0.0687	0.0660	0.2542	0.1141	0.1114	0.0909	0.0873	-0.1805	-0.1604	-0.1577
	0.75	0.1990	0.1925	0.3689	0.2869	0.2829	0.2345	0.2206	-0.3585	-0.3400	-0.3376
	0.6	0.4750	0.4662	0.5736	0.5729	0.5690	0.4975	0.4719	-0.5774	-0.5605	-0.5583

TABLE S7 Empirical rejection rates for different statistical tests of nominal size $\omega = 0.05$ when the true process has intensities $\lambda_{0k}(t|X) = \lambda_k \exp(\gamma_k X)$, along with the limiting values $\beta_{FG}^{\star}$ and $\beta_{DB}^{\star}$; γ_1 : $\log(0.9) = -0.1054, \log(0.75) = -0.2877, \log(0.6) = -0.5108$; $n = 250$ individuals, $n_{sim} = 10000$, $\tau = 1$, $P(T \leq 1|X = 0) = 0.6, P(T_1 < T_2|T \leq 1, X = 0) = 0.6, \pi_r = 0.2$.
